# The effect of empathy intervention and VR exergames on social anxiety in left-behind children

**DOI:** 10.3389/fpsyg.2025.1595174

**Published:** 2025-10-01

**Authors:** Zhiyan Xiao, Dianhui Peng, Chunxia Lu, Xueqin Zhuang

**Affiliations:** ^1^College of Physical Education, Hunan University of Science and Engineering, Yongzhou, China; ^2^College of Physical Education, Hunan Normal University, Changsha, China; ^3^College of Physical Education and Health, Linyi University, Linyi, China

**Keywords:** rural left-behind children, social anxiety, empathy intervention, VR exergames, psychological assessment

## Abstract

**Introduction:**

This study investigated the comparative effectiveness of empathy intervention and virtual-reality exergames in reducing social anxiety among rural left-behind children (RLBC) in China.

**Methods:**

Sixty RLBC from Huangdu Primary School in Shaodong City, Hunan Province were randomly assigned to three groups—the Empathy Intervention Group (EG), the VR Exergames Group (VG), and the Control Group (CG)—with 20 participants each. The Children’s Social Anxiety Scale (CSAS) was administered at baseline (T0), post-intervention (T1, Week 12), and 4-week follow-up (T2).

**Results:**

Results showed no baseline differences between groups (*P* > 0.05). At T1, both EG and VG demonstrated significant reductions in overall CSAS and subscale scores, with VG outperforming EG. By T2, VG maintained significant gains (*P* < 0.05), while EG exhibited delayed but sustained improvement (*P* < 0.05), though no significant change occurred between T1 and T2 (*P* > 0.05). The control group remained stable throughout (*P* > 0.05).

**Discussion:**

These findings suggest VR exergames yield stronger immediate effects, while empathy interventions show gradual efficacy, highlighting distinct therapeutic trajectories for RLBC’s social anxiety management.

## Introduction

With the rapid urbanization in developing countries, the scale of migrant workers has been increasing. In 2024, China’s migrant workforce totaled 299.73 million, reflecting a year-on-year increase of 2.20 million workers and a 0.7% growth rate. Among them, cross-county workers numbered 178.71 million, rising by 2.13 million workers with a 1.2% growth rate (National Bureau of Statistics, 2025). Due to institutional and economic constraints, it is hard for migrant workers to bring their children to cities where they work, resulting in a large group of left-behind children in rural China (National Bureau of Statistics, 2021). Both parents are working away from home or one parent is working away from home while the other has no guardianship ability, and the person is under the age of 16 (Ministry of Civil Affairs of the People’s Republic of China, 2016). This status confers elevated risks for Internalizing disorders and adult functional impairment (Li et al., 2021). Numerous studies have confirmed that left-behind children exhibit significantly higher levels of mental health problems compared to their peers, including elevated rates of depression and anxiety and an increased vulnerability to suicidal ideation (Fellmeth et al., 2018; Wu et al., 2019). These issues not only impede the growth and development of left-behind children but also pose a potential threat to social harmony and stability. In recent years, despite the multifaceted policy measures taken by the national government to address the lives, education, and physical and mental health issues of left-behind children, promoting their better development remains a significant challenge (Hung et al., 2023).

Notably, social anxiety disorder emerges as a critical manifestation within this complex mental health landscape. Its high prevalence and severe functional impairment distinguish it as a priority concern requiring targeted intervention (Leigh and Clark, 2018). This disorder’s profound impact on psychosocial development and its established role as a gateway to psychiatric comorbidities underscore the necessity of specialized research and clinical focus on social anxiety disorder within this vulnerable cohort.

Social anxiety represents a prevalent yet frequently overlooked psychological disorder among children (Kowalchuk et al., 2022), characterized by persistent distress and fear during real or imagined social interactions (Alvi et al., 2022). The prevalence rate of social anxiety among Chinese RLBC is 36.1%, which is significantly higher than the rate of 20.2% among non-left-behind children (Li et al., 2019). The pattern that may reflect the compounding effects of parental absence (Chen et al., 2023), reduced social support (Huang et al., 2022), and chronic interpersonal stressors inherent to their circumstances (Lin et al., 2025). This chronic condition demonstrates progressive severity and substantially elevates risks for comorbid mental health disorders, including major depressive disorder, substance use disorders, insomnia, and suicidality (Duffy et al., 2020; Kalin, 2020; Single et al., 2022). Therefore, developing targeted interventions for this vulnerable population therefore constitutes an urgent public health priority.

Empathy, one of the positive psychological qualities that has garnered widespread attention in recent years, has been shown to impact various mental health issues, including anxiety (Pittelkow et al., 2021) and depression (Liu et al., 2022). Empathy interventions with the purpose of improving individuals’ empathy, encouraging them to understand and share the feelings of others, thereby fostering better social interaction and promoting prosocial behavior. Empathy interventions hold particular relevance for RLBC due to their capacity to repair attachment disruptions caused by prolonged parental separation. RLBC tend to exhibit insecure attachment patterns (Shuang et al., 2022), and empathy training can help them rebuild their relationship patterns. Moreover, enhancing empathy contributes to interpersonal relationships and emotional regulation, which are crucial for children’s social and emotional development (Santana-Ferrándiz et al., 2025). Furthermore, VR exergames, an emerging segment within the video game industry, foster a seamless interaction between the player and the game environment. These games’ body interaction models demonstrate their compatibility with exercise (Chen et al., 2022), social interaction (Pan and Hamilton, 2018), and entertainment benefits (Hamad and Jia, 2022), thereby finding widespread applications in the health industry (Kyaw et al., 2019). The beneficiary groups span across all ages, from children and adolescents (Mittal et al., 2024) to adults (Liu et al., 2020) and even the elderly (Zahedian-Nasab et al., 2021). The immersive nature of VR exergames also makes them particularly effective for engaging children, potentially increasing adherence to therapeutic interventions (Lambert et al., 2020). Prior studies has shown that VR exergames can enhance immediate physiological regulation (Woicik et al., 2023), which is expected to bring immediate benefits to RLBC. Crucially, VR exergames offer unique advantages for rural contexts, like equipment compatibility and engagement sustainability.

In summary, amidst the growing global concern regarding the mental health challenges faced by RLBC, there remains a lack of consensus regarding the most efficacious strategies for mitigating social anxiety among this demographic. Furthermore, there is a scarcity of research investigating the impacts of empathy and VR exergames interventions on social anxiety among RLBC. Thus, this study aimed to investigate the effects of empathy and VR exergames interventions on social anxiety in RLBC, with the goal of offering enhanced insights and efficacious intervention protocols to alleviate the social anxiety issues encountered by RLBC.

## Methods

### Research design

This randomized controlled trial received ethical approval from the Biomedical Research Ethics Committee of Hunan Normal University (Approval No. 2022 No. 309). Eligible participants were stratified by baseline social anxiety score (CSAS ≥8) to ensure balanced group allocation. After completing the baseline assessment, participants were divided into three groups. Due to the distinct nature of the interventions, participants and intervention facilitators could not be blinded to group assignment. However, outcome assessors were fully blinded to group allocation throughout the study. The outcome assessors who performed the data analysis were blinded to group assignment during the initial analysis phase. Data were coded with non-identifiable group labels (Group 1, 2, 3) until the primary analyses were completed. The study was conducted in accordance with the ethical principles outlined in the Declaration of Helsinki for research involving human participants. Informed written consent was obtained from all legal guardians of participants, and written assent was additionally acquired from each child prior to their inclusion in the study. The study followed a comprehensive data collection protocol. Baseline measurements were recorded for all participants to establish pre-intervention status. Following a 12-week intervention period, the same measurements were repeated to assess the intervention’s effectiveness. A follow-up assessment was performed 4 weeks post-intervention to evaluate the sustainability of the observed effects.

### Participants

Sample size was determined *a priori* using G*Power (effect size = 0.25, *α* = 0.05, power = 0.85), yielding a required sample of 20, resulting in a total sample size of 60 participants (Faul et al., 2009; Faul et al., 2007). Participants were recruited from grades 4 and 5 at Huangdu Primary School in Shaodong City, Hunan Province. They were randomly assigned to one of three groups: the Virtual Reality Exergames group (VG), the Empathy Intervention group (EG), or the Control group (CG). Participants underwent group-specific interventions in geographically separated campus facilities, with scheduled separation to prevent cross-group interaction. The VG completed virtual reality exergames in the isolated VR classroom, while the EG received empathy training in the counseling classroom. The CG engaged in standard activities in the academic building. Strict compartmentalization of staff, scheduling, and physical spaces was maintained throughout the 12-week intervention period. All data were managed with strict confidentiality and were accessible only to the research team. Participants retained the right to withdraw from the study at any time for personal reasons. The recruitment criteria were as follows: (1) Children aged 9–10 years who scored ≥8 on the Children’s Social Anxiety Scale. (2) No use of antidepressants or anti-inflammatory medications in the past 3 months, and no history of psychiatric illnesses, cardiovascular, cerebrovascular, infectious, hematopoietic or endocrine system disorders, as well as no contraindications to exercise. (3) Voluntary participation with a commitment to complete a minimum of 3 months, and no physical or mental conditions that would hinder participation in exercise. (4) Exclusion of individuals with mental health disorders requiring medication or counseling. A total of 83 questionnaires were distributed in this study, and the final number of participants was confirmed to be 60 after eliminating invalid questionnaires and those that did not meet the recruitment criteria. All 60 randomized participants provided complete data at T0, T1 and T2 (0% missing), eliminating need for imputation. Attrition risk was minimized by giving gifts after completion.

### Intervention components

#### VR exergames group

Eligible participants were scheduled for the VR exergame intervention three times per week for 12 weeks, with each session lasting approximately 45 min. Prior to each session, a 5- to 10-min warm-up supervised by a student assistant to ensure they were physically prepared. Simultaneously, the research assistant ensured the cleanliness of the VR classroom, organized the teaching environment, and verified equipment functionality. The intervention was divided into two phases. This progression was intentionally designed to scaffold skill acquisition: the initial high-intensity boxing game (Fit XR) built foundational motor skills and engagement, while the subsequent culturally tailored aquatic game (‘Seas The Day’) introduced complex coordination in low-threat social scenarios. The shift from Oculus Quest 1 (Phase 1) to Quest 2 (Phase 2) was necessitated by the technical requirements of the custom-developed ‘Seas The Day’ software, which utilized hand-tracking features unavailable on Quest 1. It is particularly emphasized that all participants used identical device sequences, and both phases provided 45-min sessions of goal-directed aerobic exercise within immersive VR. During the initial 6 weeks, participants were assisted by an experimenter in wearing the Oculus Quest 1 headset and were instructed on playing the Fit XR sports game, which includes boxing-inspired VR fitness activities such as jabs, uppercuts, defensive maneuvers, squats, and lateral movements. For the subsequent 6 weeks, participants engaged in the customized VR sports game ‘Seas The Day’ using the Oculus Quest 2 headset. This game featured three virtual locations offering a Tai Chi warm-up, rowing physical training, and a fishing cool-down. Participants were guided through the activities by recorded audio cues, with visual-tactile prompts encouraging interaction. Interaction with virtual elements required no button presses, and players were automatically transitioned between game stages to minimize errors.

#### Empathy group

The empathy intervention was a structured, group-based program designed to enhance empathy skills (specifically emotion recognition, perspective-taking, and empathic concern) and reduce social anxiety through cognitive-behavioral techniques and experiential learning (Depow et al., 2025). Participants in the empathy intervention group received training sessions lasting 45 min each, three times a week, for a duration of 12 weeks. Sessions were conducted in small groups of 4–5 children to encourage participation and interaction. The intervention included both didactic components and active, practical exercises: (1) Didactic Introduction (weeks 1–2): Sessions began with brief psychoeducation on identifying basic emotions (happy, sad, angry, and scared) in oneself and others using facial expressions, body language, and contextual cues (using pictures and short videos). (2) Core Practical Activities (weeks 3–12): The majority of each session focused on interactive and experiential exercises. (a) Role-Playing: Children engaged in structured role-playing scenarios depicting common social interactions relevant to their age group (e.g., joining a game, asking for help, comforting a friend who is upset). Each scenario involved assigning roles, acting out the situation, and facilitated group discussion focusing on understanding the feelings and perspectives of different characters; (b) Perspective-Taking Exercises: Using stories or videos depicting social dilemmas, children were guided to explicitly articulate “How might the character be feeling?” and “Why might they feel that way?.” Group discussions encouraged considering multiple viewpoints; (c) Emotion Charades and Guessing Games: Children practiced expressing different emotions non-verbally for others to guess, and vice versa, enhancing emotion recognition skills in a playful, practical setting; (d) Group Discussions and Sharing: Facilitated discussions encouraged children to share times they felt similar emotions or encountered similar situations, fostering mutual understanding and normalization of feelings. This comprehensive approach ensured that participants not only understood empathy but also appreciated its importance and developed practical skills to exhibit empathetic behaviors.

#### Control group

The control group comprised 20 rural children left behind, randomly selected to undergo no specialized intervention over the 12-week study period. However, they received equivalent monitoring as the intervention group, ensuring consistency in data collection and observation. This control group serves as a pivotal reference point for assessing the efficacy of the diverse interventions implemented throughout the study. By comparing outcomes between the intervention and control groups, the impact of the interventions on the targeted variables can be accurately evaluated, thus enhancing the robustness and reliability of the study findings.

### Measurement

#### Demographic information questionnaire

This section gathered basic participant characteristics. Items included gender, age, sibling status, parental employment status, frequency of parental presence at home, parental marital status, parental occupation, and household income level.

#### Social anxiety

The second section utilized the Children’s Social Anxiety Scales developed by Greca et al. (1993) to assess the participants’ levels of social anxiety. This scale, applicable to children and adolescents aged 7–16, consists of 10 items (e.g., “I am afraid of doing something I have not done before in front of other children”) and covers two dimensions: Fear of Negative Evaluation (FNE) and Social Avoidance and Distress (SAD). Responses are scored on a three-point scale: never = 0, sometimes = 1, always = 2. Higher total scores indicate more severe social anxiety. According to the Chinese urban norm, a score of ≥8 indicates social anxiety (Li et al., 2006). In this study, the Cronbach’s *α* for the overall scale was 0.892, with each dimension was 0.809 (FNE), 0.825 (SAD), respectively.

### Data analysis

Statistical analysis was conducted using GraphPad Prism (9th edition), and results were presented as mean ± standard deviation (M ± SD). Shapiro–Wilk tests confirmed univariate normality. Initially, differences in baseline characteristics among the three groups were assessed using either the chi-square test or one-way ANOVA. Subsequently, a repeated-measures ANOVA, employing a 3 (group: EG, VG, CG) × 3 (time: pre-intervention, post-intervention, 4-week follow-up after intervention) design, was utilized to evaluate the effects of different intervention programs on the social anxiety levels of left-behind children. The significance level for all statistical tests was set at 0.05, with *p* < 0.05 (*) denoting statistical significance.

## Results

### Descriptive statistics

Table 1 indicated no significant differences among the three groups regarding general demographic information, including gender, age, grade, sibling status, parents’ marital and employment status, frequency of parents returning home, parental occupation, and family income (*p* > 0.05). All demographic variables in Table 1 were evaluated as potential covariates for social anxiety outcomes. None demonstrated significant group-wise differences (*p* > 0.05), confirming their homogeneity across experimental conditions.

**Table 1:** Baseline demographic characteristics by group assignment with between-group comparisons for social anxiety covariates [*M ± SD*, *n* (%)].

Variable	Characteristic	CG (*n* = 20)	EG (*n* = 20)	VG (*n* = 20)	*F*/*χ*^2^	*P*
Gender	Male	12(60.00)	11(55.00)	10(50.00)	0.19	0.817
	Female	8(40.00)	9(45.00)	10(50.00)		
Age (year)		10.60 ± 0.75	10.25 ± 0.91	10.15 ± 0.88	1.55	0.624
Single child	Single child	14(70.00)	12(60.00)	14(70.00)	0.29	0.741
	Non-single child	6(30.00)	8(40.00)	6(30.00)		
Parents’ marital status	Married	13(65.00)	12(60.00)	13(65.00)	0.07	0.996
	Divorced	4(20.00)	5(25.00)	5(25.00)		
	Remarried	2(10.00)	2(10.00)	1(5.00)		
	Widowed	1(5.00)	1(5.00)	1(5.00)		
Parents working	Father out	9(45.00)	9(45.00)	12(60.00)	0.49	0.844
	Mother out	6(30.00)	5(25.00)	4(20.00)		
	Both parents out	5(25.00)	6(30.00)	4(20.00)		
Frequency of parents returning home	1 time/week	1(5.00)	2(10.00)	2(10.00)	0.09	0.991
	1 time/month	3(15.00)	3(15.00)	2(10.00)		
	1 time/year	11(55.00)	10(50.00)	10(50.00)		
	<1 time/year	5(25.00)	5(25.00)	6(30.00)		
Parents’ occupation	Farmer	4(20.00)	3(15.00)	4(20.00)	0.19	0.979
	Work	11(55.00)	13(65.00)	13(65.00)		
	Self-employment	3(15.00)	3(15.00)	2(10.00)		
	Others	2(10.00)	1(5.00)	1(5.00)		
Family income (yuan/month)	<5,000	3(15.00)	2(10.00)	2(10.00)	0.02	0.983
	<8,000	6(30.00)	7(35.00)	6(30.00)		
	<10,000	9(47.62)	9(45.00)	11(55.00)		
	>10,000	2(9.52)	2(10.00)	1(5.00)		
SA	Total score	10.45 ± 2.01	11.35 ± 3.23	11.05 ± 3.35	0.49	0.253
	FNE	6.20 ± 1.61	6.90 ± 2.00	6.75 ± 1.94	0.79	0.453
	SAD	4.65 ± 1.22	4.80 ± 2.07	4.85 ± 1.46	0.08	0.110

Categorical variables: *χ*^2^ test; continuous variables: one-way ANOVA; SA, social anxiety; FNE, fear of negative evaluation; SAD, social avoidance and distress.

### Effects of different interventions on social anxiety

**Figure 1:**
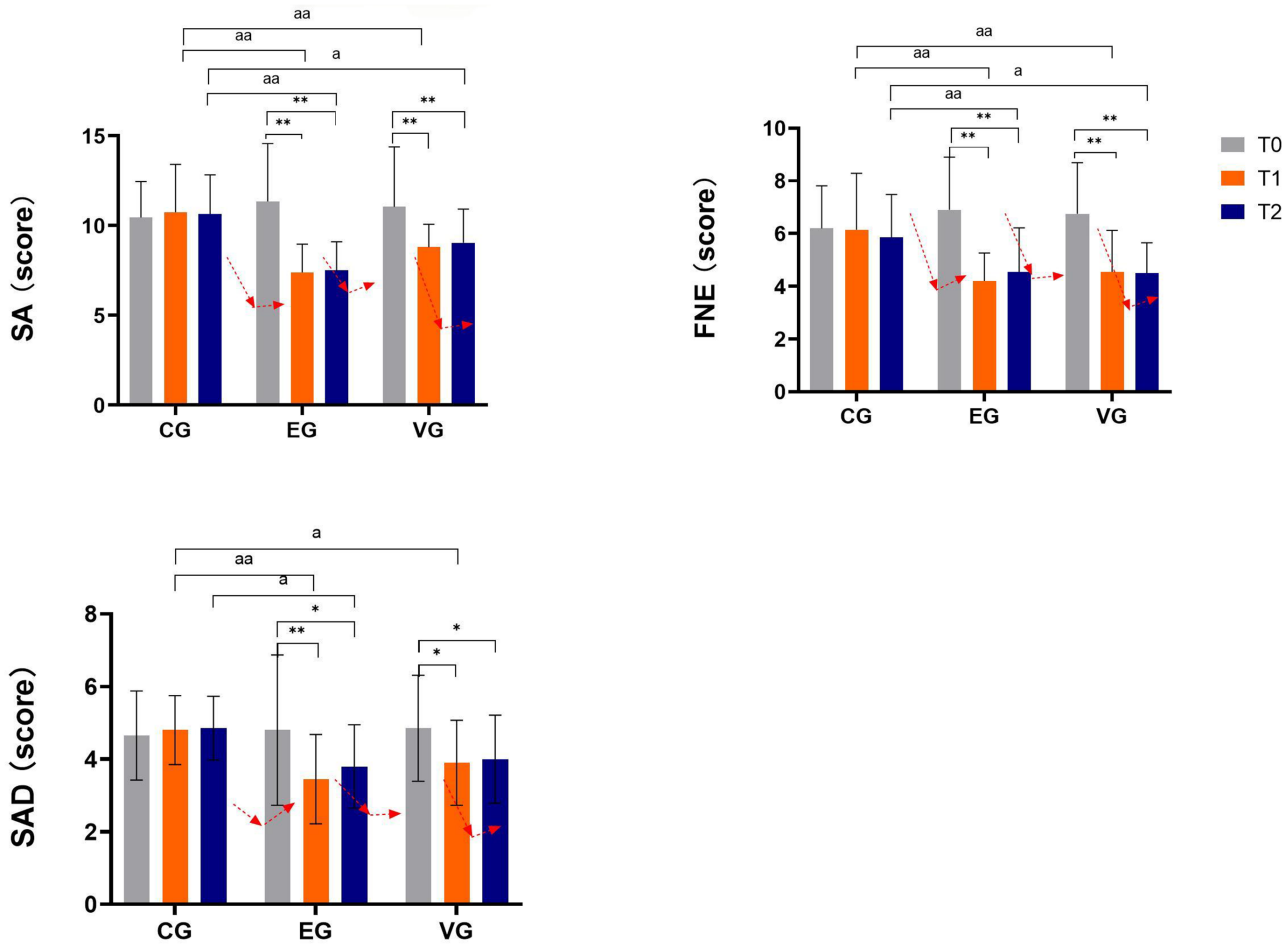
Effects of different interventions on SA, FNE, SAD. * *p* <0.05, ** *p*<0.01. a < 0.05, aa < 0.01, compared with CG.

Comprehensive data regarding social anxiety scores and its two dimensions were analyzed both at baseline and upon completion of the intervention across three distinct groups. This investigation aimed to delineate the impact of various interventions on the social anxiety levels among RLBC. Following rigorous statistical analysis, a notable cluster-time interaction emerged concerning the social anxiety scale [*F*(4,57) = 6.70, *p* < 0.01, *η^2^* = 0.19], underscoring the intervention’s effect over time. Additionally, a significant interaction surfaced between the FNE factor [*F*(4,57) = 4.70, *p* < 0.01, *η^2^* = 0.14] and SAD [*F*(4,57) = 2.53, *p* < 0.01, *η^2^* = 0.08], suggesting a nuanced relationship between these dimensions. To further elucidate these findings, subsequent simple effect analyses were conducted. Results displayed in Figure 1 demonstrate a consistent decrease in social anxiety levels and factor scores across intervention groups, correlating with the intervention duration. Importantly, a notable divergence between the intervention groups was observed, signifying the differential efficacy of interventions in mitigating social anxiety among the studied population.

Our investigation into time-related factors revealed significant variations in the total score [*F*(1,57) = 17.10, *p* < 0.01, *η^2^* = 0.23], FNE factor [*F*(1,57) = 22.85, *p* < 0.01, *η^2^* = 0.29], and SAD factor [*F*(1,57) = 6.05, *p* < 0.01, *η^2^* = 0.96] over time. Notably, CG displayed no significant change in total score, FNE, and SAD dimensions over time (*p* > 0.05), indicating relative stability in social anxiety levels throughout the intervention. Conversely, EG and VG exhibited statistically significant reductions in total scores, FNE, and SAD scores at T1 and T2 compared to T0 (*p* < 0.05), suggesting a positive impact of interventions on diminishing social anxiety levels within these groups.

In the group factor analysis, significant effects were observed for the total score [*F*(2,57) = 7.20, *p* < 0.01, *η^2^* = 0.20], FNE factor [*F*(2,57) = 3.74, *p* < 0.01, *η^2^* = 0.12], and SAD factor [*F*(2,57) = 3.85, *p* < 0.01, *η^2^* = 0.12]. Initially, at baseline (T0), no significant differences existed between intervention and control groups in total score, FNE, and SAD factors (*p* > 0.05). However, during the intervention, CG showed significantly higher total scores than the other two groups (*p* < 0.05). VG had notably higher scores than EG at both T1 and T2 (*p* < 0.05). Regarding FNE scores, CG scored significantly higher than the other two groups at both T1 and T2 (*p* < 0.05). Nonetheless, EG scored higher than VG at T1 (*p* > 0.05), with no significant difference between the two groups at T1 and T2. For SAD scores, CG scored significantly higher than the other two groups at T1 (*p* < 0.05). Importantly, no significant differences were found between VG and EG in total scores and scores across all dimensions (*p* > 0.05), although EG scored higher than VG at T1 and T2 (Table 2 and Figure 1).

**Table 2:** Effects of different interventions on SA, FNE and SAD (*M ± SD*).

Variables	Group	T0	T1	T2	*F*/*η*^2^/*P*_group_	*F*/*η*^2^/*P*_time_	*F*/*η*^2^/*P*_group*time_
SA	CG	10.45 ± 2.01	10.75 ± 2.67	10.65 ± 2.18	7.20/0.20*/*<0.001^***^	17.1/0.23*/*<0.001^***^	6.70/0.19*/*<0.01^**^
	VG	11.35 ± 3.23	7.40 ± 1.57^aa**^	7.50 ± 1.61^aa**^			
	EG	11.05 ± 3.35	8.80 ± 1.28^aa*^	9.05 ± 1.88^a*^			
FNE	CG	6.20 ± 1.61	6.15 ± 2.13	5.85 ± 1.63	3.74/0.12*/*<0.001^***^	22.85/0.29*/*<0.001^***^	4.70/0.14*/*<0.01^**^
	VG	6.90 ± 2.00	4.20 ± 1.06^aa**^	4.55 ± 1.67^aa**^			
	EG	6.75 ± 1.94	4.55 ± 1.57^aa**^	4.50 ± 1.15^a**^			
SAD	CG	4.65 ± 1.23	4.80 ± 0.95	4.85 ± 0.88	3.85/0.12*/*<0.001^***^	6.05/0.10*/*<0.001^**^	2.53/0.08*/*<0.01^**^
	VG	4.80 ± 2.07	3.45 ± 1.23^aa**^	3.80 ± 1.15^a*^			
	EG	4.85 ± 1.46	3.90 ± 1.17^a*^	4.00 ± 1.21^*^			

a < 0.05, aa<0.01, compared with CG; ^*^P < 0.05, ^**^P < 0.01, ^***^P < 0.001, compared with T0; SA, social anxiety; FNE, fear of negative evaluation; SAD, social avoidance and distress.

## Discussion

This study aimed to explore the impact of empathy intervention and VR exergames on social anxiety among RLBC, drawing from a sample of 60 participants. Results revealed significant reductions in social anxiety following interventions with both empathy intervention and VR exergames. However, the effectiveness of these interventions is influenced by multifaceted factors, including cognitive and humanistic aspects inherent in empathy, as well as the characteristics of movement, such as intensity, duration, and content, found in VR exergames. While both interventions significantly reduced social anxiety in RLBC, our repeated-measures ANOVA revealed a moderately stronger treatment effect for VG compared to EG. In particular, post tests showed no significant difference between EG and VG at T2, suggesting comparable short-term efficacy despite divergent change trajectories. VG’s advantage was driven by rapid social avoidance reduction, and EG showed progressive fear of negative evaluation improvements. VG offers accelerated symptom relief for acutely avoidant RLBC, whereas EG may benefit youth with predominant evaluative fears—supporting personalized intervention matching. This observation underscores the nuanced impact of different interventions on social anxiety among RLBC. Strict exclusion (psychiatric comorbidities) enhances internal validity but reduces applicability to clinical RLBC populations. Effectiveness trials with stepped-care approaches are needed in the future. In addition, future research can consider the combination of the two to form a long-term intervention mechanism and obtain long-term efficacy. Further exploration into the nuanced effects of these interventions could provide valuable insights into tailored approaches for addressing social anxiety in this population.

Empathy and social anxiety demonstrate an inverse relationship, where an increase in empathy levels correlates with a reduction in social anxiety, consistent with prior research (Li et al., 2024). Theoretical models highlight impaired emotion regulation as a significant factor in social anxiety development and maintenance (Daniel et al., 2019; Daniel et al., 2023). Individuals with social anxiety often struggle to recognize, understand, and tolerate negative emotions in others, suggesting poor emotion regulation as a common underlying mechanism (Sackl-Pammer et al., 2019). Empathy, viewed as a form of emotion regulation, has been identified as a predictor of social anxiety, prompting this study to replicate and extend previous findings (Tan et al., 2023). Gilbert’s model of empathy development posits that empathy entails the capacity for self-soothing during distress and the suppression of self-criticism, traits influenced by early attachment experiences with caregivers (Gilbert, 2020). Secure attachment, fostered by consistent parental support and environmental control, lays the foundation for self-compassion in adulthood and facilitates both giving and receiving empathy (Calandri et al., 2019). Conversely, ineffective parenting, prevalent among individuals with social anxiety, undermines these abilities (Vietmeier et al., 2025). Empirical evidence links empathy with social anxiety and indicates that interventions targeting empathy can alleviate social anxiety symptoms. For instance, Werner utilized Neff’s self-compassion measure and found lower self-compassion levels in clinically diagnosed individuals with social anxiety compared to those without psychiatric conditions (Werner et al., 2012). Moreover, Rebecca revealed that self-compassion correlated with heightened *post hoc* processing in socially anxious individuals marked by chronic negative rumination (Blackie and Kocovski, 2018). Neuroimaging studies support these findings, showing increased medial prefrontal cortex activation during rumination tasks, underscoring the cognitive mechanisms underlying social anxiety (Do Bú et al., 2022; Watkins and Roberts, 2020). The robustness of the relationship between empathy and social anxiety is underscored by empirical evidence demonstrating that interventions aimed at fostering empathy can mitigate this post-processing tendency and alleviate symptoms of social anxiety (Tang et al., 2024). While slower-acting, empathy interventions uniquely address attachment trauma from prolonged separation. Relevant departments and schools can promote the inclusion of empathy training in psychology classes.

VR exergames and social anxiety demonstrate an inverse relationship, a condition that can be mitigated through targeted VR exergames interventions. Recent research underscores the efficacy of employing VR exergames to ameliorate symptoms associated with anxiety disorders and to bolster anxiety prevention strategies (Freeman et al., 2017). Notably, VR exergames integrating exercise and recreational elements have emerged as promising avenues for inducing anxiolytic effects. Exercise has been demonstrated to alter the monoamine levels (e.g., serotonin, dopamine, and norepinephrine) and stress hormone cortisol to maintain mood (Fossati et al., 2021; Zong et al., 2022). On the other hand, there is some evidence showing that exercise can promote neuroprotection through molecular adaptations (e.g., the activation of the PGC-1α/FNDC5/Irisin pathway), leading to maintaining good mental health (Dastamooz et al., 2023). Further elaboration on the specific methodologies and mechanisms underlying the efficacy of VR exergames interventions could enhance our understanding of their therapeutic potential in addressing social anxiety. This effect may stem from several factors inherent to VR exergames. Firstly, the social nature of these games, provides ample opportunity for social interaction, which can influence the formation of friendships, bolster self-esteem, regulate mood, and enhance motivation, consequently augmenting real-life social behaviors (Meins et al., 2023). Additionally, certain VR exergames incorporate visual or auditory stimuli to simulate virtual environments. For instance, auditory elements such as music engage a myriad of brain structures and neurotransmitter systems associated with rewards, motivation, pleasure, and stress regulation (Liu et al., 2025). While music or auditory elements were present in some VR modules, our design did not isolate these components to attribute effects. Future studies should dissect whether auditory stimuli independently contribute to anxiety reduction in this population. Moreover, the immersive virtual environments provided by VR exergames have been shown to induce positive emotions and mitigate negative emotions and anxiety in individuals (Jiang et al., 2024). These findings align with recent research indicating that virtual reality video games elicit positive emotional responses in players (Maggio et al., 2024). This underscores the potential of VR exergames as a viable adjunct or alternative therapeutic approach for individuals struggling with anxiety disorders. The superior immediate efficacy of VR addresses RLBC’s crisis-level need for accessible interventions. Unlike clinic-based therapies requiring specialists, mobile VR systems are easy to operate and can be used formally by teachers through training. Therefore, VR can be considered to be introduced into daily teaching. For example, schools in less developed rural areas do not have the conditions to have physical education classes outdoors on rainy days. At present, cheap basic VR equipment can meet the needs of teaching and promote the physical and mental health development of children.

### Strengths and limitations

In this study, interventions were delivered within the children’s school setting, increasing practical applicability. Moreover, examination of both overall social anxiety and its subdomains provided nuanced insights. However, the study still has some limitations. Firstly, while this study utilized self-report measures to assess common behavioral patterns, it is important to note the limitations of such measures. Self-report instruments may not always provide an accurate description of behavior in social settings and can be influenced by participant response biases. To enhance the robustness of findings, future research endeavors could integrate objective methodologies, such as observing physiological responses and measuring behavioral performance, to better capture participants behavior in social environment. Subsequently, the 4-week post-intervention assessment, while sufficient to capture immediate treatment effects, is insufficient to evaluate long-term efficacy. Given school semester schedule constraints and the scarcity of RLBC research, building proof of concept precedes vertical investment. Longer follow-up can be performed in future studies. Finally, the study’s reliance on a relatively small sample size of elementary school students from a specific city in China, along with stringent recruitment criteria for socially anxious individuals, poses limitations on the generalizability of results. However, as an earlier RCT in RLBC, pragmatic constraints (e.g., limited eligible schools) necessitated focused sampling. Considering the predictable social conditions and challenges influenced by the geographical and cultural backgrounds of the participants, it becomes imperative to expand the scope of investigation. Future studies should aim to include RLBC from diverse geographical and cultural backgrounds to improve the generalizability of findings and enrich the understanding of their social experiences.

## Conclusion

This study aims to explore the impact of empathy intervention and VR exergames on social anxiety among left-behind children. Through comparative analysis, we found that both empathy training and VR exergames could significantly reduce the level of social anxiety in this population, and VR intervention was more significant. For RLBC with attachment disruptions, empathy training may rebuild mentalizing capacity damaged by caregiver absence, while VR exergames compensate for underdeveloped body awareness through enforced interoceptive exposure. This outcome not only validates the efficacy of empathy training and VR exergames as a therapeutic intervention for alleviating social anxiety in left-behind children but also introduces a novel therapeutic avenue to address their mental health needs. Consequently, advocating for integrating empathy training and VR exergames intervention strategies into future clinical practices can potentially ameliorate social anxiety among left-behind children, offering a more robust foundation for informed clinical decision-making.

## Data Availability

The raw data supporting the conclusions of this article will be made available by the authors, without undue reservation.

## References

[ref1] Alvi T. Kumar D. Tabak B. A. (2022). Social anxiety and behavioral assessments of social cognition: a systematic review. J. Affect. Disord. 311, 17–30. doi: 10.1016/j.jad.2022.04.130, PMID: 35490878 PMC9754122

[ref2] Blackie R. A. Kocovski N. L. (2018). Examining the relationships among self-compassion, social anxiety, and post-event processing. Psychol. Rep. 121, 669–689. doi: 10.1177/0033294117740138, PMID: 29298554

[ref3] Calandri E. Graziano F. Testa S. Cattelino E. Begotti T. (2019). Empathy and depression among early adolescents: the moderating role of parental support. Front. Psychol. 10:1447. doi: 10.3389/fpsyg.2019.01447, PMID: 31316426 PMC6610578

[ref4] Chen J. Or C. K. Chen T. (2022). Effectiveness of using virtual reality-supported exercise therapy for upper extremity motor rehabilitation in patients with stroke: systematic review and Meta-analysis of randomized controlled trials. J. Med. Internet Res. 24:e24111. doi: 10.2196/24111, PMID: 35723907 PMC9253973

[ref5] Chen L. Wulczyn F. Huhr S. (2023). Parental absence, early reading, and human capital formation for rural children in China. J. Community Psychol. 51, 662–675. doi: 10.1002/jcop.22786, PMID: 34985781

[ref6] Daniel K. E. Baee S. Boukhechba M. Barnes L. E. Teachman B. A. (2019). Do I really feel better? Effectiveness of emotion regulation strategies depends on the measure and social anxiety. Depress. Anxiety 36, 1182–1190. doi: 10.1002/da.22970, PMID: 31652383

[ref7] Daniel K. E. Larrazabal M. A. Boukhechba M. Barnes L. Teachman B. A. (2023). State and trait emotion regulation diversity in social anxiety. Clin. Psychol. Sci. 11, 894–909. doi: 10.1177/21677026231151956, PMID: 37981951 PMC10656041

[ref8] Dastamooz S. Sadeghi-Bahmani D. Farahani M. H. D. Wong S. H. S. Yam J. C. S. Tham C. C. Y.. (2023). The efficacy of physical exercise interventions on mental health, cognitive function, and ADHD symptoms in children and adolescents with ADHD: an umbrella review. EClinicalMedicine 62:102137. doi: 10.1016/j.eclinm.2023.102137, PMID: 37599910 PMC10432969

[ref9] Depow G. J. Oldemburgo de Mello V. Inzlicht M. (2025). A positive empathy intervention to improve well-being on Instagram. Emotion 25, 1207–1224. doi: 10.1037/emo0001489 39883419

[ref10] Do Bú E. A. Santos V. M. D. Lima K. S. Pereira C. R. Alexandre M. E. S. Bezerra V. (2022). Neuroticism, stress, and rumination in anxiety and depression of people with vitiligo: an explanatory model. Acta Psychol. 227:103613. doi: 10.1016/j.actpsy.2022.103613, PMID: 35569205

[ref11] Duffy M. E. Mueller N. E. Cougle J. R. Joiner T. E. (2020). Perceived burdensomeness uniquely accounts for suicidal ideation severity in social anxiety disorder. J. Affect. Disord. 266, 43–48. doi: 10.1016/j.jad.2020.01.116, PMID: 32056911

[ref12] Faul F. Erdfelder E. Buchner A. Lang A. G. (2009). Statistical power analyses using G*power 3.1: tests for correlation and regression analyses. Behav. Res. Methods 41, 1149–1160. doi: 10.3758/brm.41.4.1149, PMID: 19897823

[ref13] Faul F. Erdfelder E. Lang A. G. Buchner A. (2007). G*power 3: a flexible statistical power analysis program for the social, behavioral, and biomedical sciences. Behav. Res. Methods 39, 175–191. doi: 10.3758/bf03193146, PMID: 17695343

[ref14] Fellmeth G. Rose-Clarke K. Zhao C. Busert L. K. Zheng Y. Massazza A.. (2018). Health impacts of parental migration on left-behind children and adolescents: a systematic review and meta-analysis. Lancet 392, 2567–2582. doi: 10.1016/s0140-6736(18)32558-3, PMID: 30528471 PMC6294734

[ref15] Fossati C. Torre G. Vasta S. Giombini A. Quaranta F. Papalia R.. (2021). Physical exercise and mental health: the routes of a reciprocal relation. Int. J. Environ. Res. Public Health 18:12364. doi: 10.3390/ijerph182312364, PMID: 34886090 PMC8656946

[ref16] Freeman D. Reeve S. Robinson A. Ehlers A. Clark D. Spanlang B.. (2017). Virtual reality in the assessment, understanding, and treatment of mental health disorders. Psychol. Med. 47, 2393–2400. doi: 10.1017/s003329171700040x, PMID: 28325167 PMC5964457

[ref17] Gilbert P. (2020). Creating a compassionate world: addressing the conflicts between sharing and caring versus controlling and holding evolved strategies. Front. Psychol. 11:582090. doi: 10.3389/fpsyg.2020.582090, PMID: 33643109 PMC7902494

[ref18] Greca A. M. L. Stone W. L. J. J. o. C. C. Psychology, A. (1993). Social anxiety scale for children-revised: factor structure and concurrent validity. J. Clin. Child Psychol. 22, 17–27.

[ref19] Hamad A. Jia B. (2022). How virtual reality technology has changed our lives: an overview of the current and potential applications and limitations. Int. J. Environ. Res. Public Health 19:1278. doi: 10.3390/ijerph191811278, PMID: 36141551 PMC9517547

[ref20] Huang H. Wan X. Liang Y. Zhang Y. Peng Q. Ding Y.. (2022). Correlations between social support and loneliness, self-esteem, and resilience among left-behind children in mainland China: a meta-analysis. Front. Psych. 13:874905. doi: 10.3389/fpsyt.2022.874905, PMID: 35573330 PMC9095419

[ref21] Hung J. Chen J. Chen O. (2023). The practice of social protection policies in China: a systematic review on how left-behind children's mental health can be optimised. Perspect. Public Health 145:17579139231205491. doi: 10.1177/17579139231205491, PMID: 37889069

[ref22] Jiang W. Windl M. Tag B. Sarsenbayeva Z. Mayer S. (2024). An immersive and interactive VR dataset to elicit emotions. IEEE Trans. Vis. Comput. Graph. 30, 7343–7353. doi: 10.1109/tvcg.2024.3456202, PMID: 39255143

[ref23] Kalin N. H. (2020). The critical relationship between anxiety and depression. Am. J. Psychiatry 177, 365–367. doi: 10.1176/appi.ajp.2020.20030305 32354270

[ref24] Kowalchuk A. Gonzalez S. J. Zoorob R. J. (2022). Anxiety disorders in children and adolescents. Am. Fam. Physician 106, 657–664. 36521463

[ref25] Kyaw B. M. Saxena N. Posadzki P. Vseteckova J. Nikolaou C. K. George P. P.. (2019). Virtual reality for health professions education: systematic review and meta-analysis by the digital health education collaboration. J. Med. Internet Res. 21:e12959. doi: 10.2196/12959, PMID: 30668519 PMC6362387

[ref26] Lambert V. Boylan P. Boran L. Hicks P. Kirubakaran R. Devane D.. (2020). Virtual reality distraction for acute pain in children. Cochrane Database Syst. Rev. 10:Cd010686. doi: 10.1002/14651858.CD010686.pub2, PMID: 33089901 PMC8094164

[ref27] Leigh E. Clark D. M. (2018). Understanding social anxiety disorder in adolescents and improving treatment outcomes: applying the cognitive model of Clark and Wells (1995). Clin. Child. Fam. Psychol. Rev. 21, 388–414. doi: 10.1007/s10567-018-0258-5, PMID: 29654442 PMC6447508

[ref28] Li X. Peng D. Wu X. Li X. Liang J. Yin H.. (2024). Effects of empathy on loneliness among rural left-behind children in China: the chain-mediated roles of social anxiety and psychological resilience. Psychol. Res. Behav. Manag. 17, 3369–3379. doi: 10.2147/prbm.S477556, PMID: 39371939 PMC11451400

[ref29] Li M. L. Ren Y. J. Jiang F. (2019). A meta-analysis of social anxiety in left-behind children in rural areas of China. J. Chin. Ment. Health 33, 839–844.

[ref30] Li F. Su L.-Y. Jin Y. Children, N. C. G. O. A. S. F (2006). Chinese urban norm of social anxiety scale for children. Chinese Journal of Child Health Care 4, 335–337.

[ref31] Li H. M. Xu Y. M. Zhong B. L. (2021). Relationship between childhood left-behind experience and quality of life among Chinese university freshmen: place of origin matters. Front. Psych. 12:789622. doi: 10.3389/fpsyt.2021.789622, PMID: 34899441 PMC8651710

[ref32] Lin K. Mak L. Cai J. Jiang S. Fayyaz N. Broadley S.. (2025). Urbanisation and mental health in left-behind children: systematic review and meta-analysis using resilience framework. Pediatr. Res. doi: 10.1038/s41390-025-03894-5, PMID: 39910352 PMC12507658

[ref33] Liu G. Hu J. Kostikova I. (2025). Music therapy and its impact on anxiety and mental well-being of Chinese students: an experimental comparison of traditional and VR approaches. Acta Psychol. 255:104898. doi: 10.1016/j.actpsy.2025.104898, PMID: 40106973

[ref34] Liu Y. Song Y. Tamura R. (2020). Hedonic and utilitarian motivations of home motion-sensing game play behavior in China: an empirical study. Int. J. Environ. Res. Public Health 17:8794. doi: 10.3390/ijerph17238794, PMID: 33256148 PMC7730092

[ref35] Liu Q. Zhao X. Liu W. Liu Q. (2022). Empathy and depression among a Chinese sample: the moderating role of rumination and attentional shift. Front. Psychol. 13:1027298. doi: 10.3389/fpsyg.2022.1027298, PMID: 36507000 PMC9729700

[ref36] Maggio M. G. Benenati A. Impellizzeri F. Rizzo A. Barbera M. Cannavò A.. (2024). Impact of cognitive VR vs. traditional training on emotional self-efficacy and cognitive function in patients with multiple sclerosis: a retrospective study focusing on gender differences. Brain Sci. 14:1227. doi: 10.3390/brainsci14121227, PMID: 39766426 PMC11674292

[ref37] Meins I. A. Muijsson-Bouwman D. C. Nijman S. A. Greaves-Lord K. Veling W. Pijnenborg G. H. M.. (2023). VR-SOAP, a modular virtual reality treatment for improving social activities and participation of young people with psychosis: a study protocol for a single-blind multi-Centre randomized controlled trial. Trials 24:278. doi: 10.1186/s13063-023-07241-z, PMID: 37061694 PMC10105944

[ref38] Ministry of Civil Affairs of the People’s Republic of China. (2016). A responsible person from the Ministry of Civil Affairs answered questions from reporters regarding the situation of the census and investigation of left-behind children in rural areas. Available online at: https://www.mca.gov.cn/n152/n164/c35255/content.html (accessed July, 2025)

[ref39] Mittal P. Bhadania M. Tondak N. Ajmera P. Yadav S. Kukreti A.. (2024). Effect of immersive virtual reality-based training on cognitive, social, and emotional skills in children and adolescents with autism spectrum disorder: a meta-analysis of randomized controlled trials. Res. Dev. Disabil. 151:104771. doi: 10.1016/j.ridd.2024.104771, PMID: 38941690

[ref40] National Bureau of Statistics. (2021) Seventh national population census bulletin (No. 7) https://www.stats.gov.cn/sj/zxfb/202302/t20230203_1901087.html (accessed July, 2025).

[ref41] National Bureau of Statistics. (2025). Report on the monitoring and survey of migrant workers in 2024

[ref42] Pan X. Hamilton A. F. C. (2018). Why and how to use virtual reality to study human social interaction: the challenges of exploring a new research landscape. Br. J. Psychol. 109, 395–417. doi: 10.1111/bjop.12290 29504117 PMC6055846

[ref43] Pittelkow M. M. Het Rot M. Aan Seidel L. J. Feyel N. Roest A. M. Social anxiety and empathy: a systematic review and meta-analysis J. Anxiety Disord. (2021). 78:102357 doi: 10.1016/j.janxdis.2021.102357 PMID: 33588287

[ref44] Sackl-Pammer P. Jahn R. Özlü-Erkilic Z. Pollak E. Ohmann S. Schwarzenberg J.. (2019). Social anxiety disorder and emotion regulation problems in adolescents. Child Adolesc. Psychiatry Ment. Health 13:37. doi: 10.1186/s13034-019-0297-9, PMID: 31583014 PMC6771087

[ref45] Santana-Ferrándiz M. Ibáñez-Pérez J. Moret-Tatay C. (2025). Empathy and parental sensitivity in child attachment and socioemotional development: a systematic review from emotional, genetic, and neurobiological perspectives. Children 12:465. doi: 10.3390/children12040465, PMID: 40310085 PMC12025558

[ref46] Shuang M. Yiqing W. Ling J. Guanzhen O. Jing G. Zhiyong Q.. (2022). Relationship between parent-child attachment and depression among migrant children and left-behind children in China. Public Health 204, 1–8. doi: 10.1016/j.puhe.2021.12.015, PMID: 35065353

[ref47] Single A. Bilevicius E. Ho V. Theule J. Buckner J. D. Mota N.. (2022). Cannabis use and social anxiety in young adulthood: a meta-analysis. Addict. Behav. 129:107275. doi: 10.1016/j.addbeh.2022.107275, PMID: 35184002

[ref48] Tan X. Yang Y. Yu M. (2023). Longitudinal relationship of empathy and social anxiety among adolescents: the mediation roles of psychological inflexibility and rejection sensitivity. J. Affect. Disord. 339, 867–876. doi: 10.1016/j.jad.2023.07.069, PMID: 37467804

[ref49] Tang Q. Zou X. Li Y. Xu Y. Lv Y. Liu X.. (2024). Insomnia mediates the relation between empathy and anxiety among nursing students: a latent moderated mediation model of self-compassion. BMC Nurs. 23:570. doi: 10.1186/s12912-024-02238-8, PMID: 39152435 PMC11330050

[ref50] Vietmeier N. Tuschen-Caffier B. Asbrand J. (2025). Social stress task with parental support or self-instruction decreases negative cognitions in children with social anxiety disorder. Sci. Rep. 15:10220. doi: 10.1038/s41598-025-95032-8, PMID: 40133558 PMC11937552

[ref51] Watkins E. R. Roberts H. (2020). Reflecting on rumination: consequences, causes, mechanisms and treatment of rumination. Behav. Res. Ther. 127:103573. doi: 10.1016/j.brat.2020.103573 32087393

[ref52] Werner K. H. Jazaieri H. Goldin P. R. Ziv M. Heimberg R. G. Gross J. J. (2012). Self-compassion and social anxiety disorder. Anxiety Stress Coping 25, 543–558. doi: 10.1080/10615806.2011.608842, PMID: 21895450 PMC4128472

[ref53] Woicik K. Geraets C. N. W. Klein Tuente S. Masthoff E. Veling W. (2023). Virtual reality aggression prevention treatment in a Dutch prison-based population: a pilot study. Front. Psychol. 14:1235808. doi: 10.3389/fpsyg.2023.1235808, PMID: 38034305 PMC10683795

[ref54] Wu W. Qu G. Wang L. Tang X. Sun Y. H. (2019). Meta-analysis of the mental health status of left-behind children in China. J. Paediatr. Child Health 55, 260–270. doi: 10.1111/jpc.14349, PMID: 30604503

[ref55] Zahedian-Nasab N. Jaberi A. Shirazi F. Kavousipor S. (2021). Effect of virtual reality exercises on balance and fall in elderly people with fall risk: a randomized controlled trial. BMC Geriatr. 21:509. doi: 10.1186/s12877-021-02462-w, PMID: 34563120 PMC8465759

[ref56] Zong B. Yu F. Zhang X. Zhao W. Sun P. Li S.. (2022). Understanding how physical exercise improves Alzheimer's disease: cholinergic and monoaminergic systems. Front. Aging Neurosci. 14:869507. doi: 10.3389/fnagi.2022.869507, PMID: 35663578 PMC9158463

